# Laquinimod ameliorates excitotoxic damage by regulating glutamate re-uptake

**DOI:** 10.1186/s12974-017-1048-6

**Published:** 2018-01-05

**Authors:** Antonietta Gentile, Alessandra Musella, Francesca De Vito, Diego Fresegna, Silvia Bullitta, Francesca Romana Rizzo, Diego Centonze, Georgia Mandolesi

**Affiliations:** 1 Unit of Neurology and Unit of Neurorehabilitation, IRCCS Istituto Neurologico Mediterraneo (INM) Neuromed, 86077 Pozzilli, IS Italy; 2 Department of Systems Medicine, Multiple Sclerosis Research Center, Tor Vergata University, Via Montpellier, 1, 00133 Rome, Italy; 3 Laboratory of Neuroimmunology and Synaptic Plasticity, Centro Europeo per la Ricerca sul Cervello (CERC), IRCCS Fondazione Santa Lucia, 00143 Rome, Italy; 4 Present address: IRCCS San Raffaele Pisana, 00163 Rome, Italy

**Keywords:** Excitotoxicity, Glutamate transporters, Experimental autoimmune encephalomyelitis, Cerebellum, Spontaneous excitatory post-synaptic currents, Neuroprotection

## Abstract

**Background:**

Laquinimod is an immunomodulatory drug under clinical investigation for the treatment of the progressive form of multiple sclerosis (MS) with both anti-inflammatory and neuroprotective effects. Excitotoxicity, a prominent pathophysiological feature of MS and of its animal model, experimental autoimmune encephalomyelitis (EAE), involves glutamate transporter (GluT) dysfunction in glial cells.

The aim of this study was to assess whether laquinimod might exert direct neuroprotective effects by interfering with the mechanisms of excitotoxicity linked to GluT function impairments in EAE.

**Methods:**

Osmotic minipumps allowing continuous intracerebroventricular (icv) infusion of laquinimod for 4 weeks were implanted into C57BL/6 mice before EAE induction. EAE cerebella were taken to perform western blot and qPCR experiments. For ex vivo experiments, EAE cerebellar slices were incubated with laquinimod before performing electrophysiology, western blot, and qPCR.

**Results:**

In vivo treatment with laquinimod attenuated EAE clinical score at the peak of the disease, without remarkable effects on inflammatory markers. In vitro application of laquinimod to EAE cerebellar slices prevented EAE-linked glutamatergic alterations without mitigating astrogliosis and inflammation. Moreover, such treatment induced an increase of Slcla3 mRNA coding for the glial glutamate–aspartate transporter (GLAST) without affecting the protein content. Concomitantly, laquinimod significantly increased the levels of the glial glutamate transporter 1 (GLT-1) protein and pharmacological blockade of GLT-1 function fully abolished laquinimod anti-excitotoxic effect.

**Conclusions:**

Overall, our results suggest that laquinimod protects against glutamate excitotoxicity of the cerebellum of EAE mice by bursting the expression of glial glutamate transporters, independently of its anti-inflammatory effects.

## Background

Multiple sclerosis (MS) is a chronic immune-mediated disease of the central nervous system (CNS) characterized by demyelination and neurodegeneration. Nowadays, neurodegeneration is not only viewed as the culminating event of demyelination but is likely supposed to develop in parallel [1]. Currently approved therapies for MS are based on immunomodulatory or immunosuppressive drugs, although effective therapies are expected to interact directly with the CNS and prevent deterioration, reverse injury, and restore function to counteract not only demyelination but also both axonal damage and synaptopathy [2–4].

Both clinical [5–7] and experimental studies in the mouse model of MS, experimental autoimmune encephalomyelitis (EAE), highlighted anti-inflammatory and neuroprotective effects of laquinimod, a once daily immunomodulatory agent under clinical investigation for the progressive form of MS [8–16]. Laquinimod reduced the number of active lesions [17, 18] and prevented the reduction in brain volume in MS patients [5]. Furthermore, laquinimod increased levels of brain-derived neurotrophic factor (BDNF) in the serum of MS patients [15] and in the CNS of EAE mice in association with a reduced CNS injury [8]. Interestingly, laquinimod, which can cross the blood–brain barrier [19], was reported to influence the viability of neurons and oligodendrocytes independently of its anti-inflammatory effects. Consistently, laquinimod reduced demyelination, axonal damage, and lesion size in EAE [8, 20] and compensated for the altered glutamatergic and GABAergic transmission in the EAE striatum [14]. Recently, it has been demonstrated that the aryl hydrocarbon receptor (AhR) is a molecular target of laquinimod in the EAE model [9].

The mechanisms by which laquinimod exerts its effects are likely different and not yet fully elucidated. In particular, little is known about its direct activity on excitotoxic damage. In MS and EAE, excitotoxicity, which involves glutamate transporter (GluT) dysfunction, is an important link between neuroinflammation and neurodegeneration [1, 21–24]. In this regard, we recently provided evidence of a defective glutamate uptake and excitotoxic damage in the EAE cerebellum [25], highlighting a dysfunction of the glial glutamate–aspartate transporter (GLAST; excitatory amino acid transporter EAAT 1 in humans) at the level of the Purkinje cell tripartite synapse. GLAST dysfunction is caused by a post-transcriptional downregulation mediated by miR-142-3-p [26].

Based on these observations, by performing ex vivo and in vivo studies in EAE mice, we investigated the direct effect of laquinimod on cerebellar glutamatergic excitotoxicity to elucidate the molecular mechanisms responsible for its neuroprotective effects.

## Methods

### Animals

Animals employed in this study were 7- to 8-week-old female mice, C57BL/6N, obtained from Charles-River (Italy) and CNR-EMMA Mouse Clinic facility (Monterotondo-Rome, Italy). Animals were randomly assigned to standard cages, with 4–5 animals per cage, and kept under standard housing conditions with a light/dark cycle of 12 h and free access to food and water. Minipump-implanted mice were housed in individual cages endowed with special bedding (TEK-FRESCH, Harlan) in order to avoid skin infections around the surgical scar.

Experiments were carried out in accordance with the Internal Institutional Review Committee, the European Directive 2010/63/EU and the European Recommendations 526/2007, and the Italian D.Lgs 26/2014. All efforts were made to minimize the number of animals used, as well as their suffering.

### EAE model

EAE was induced as previously described [25]. Mice were injected subcutaneously at the flanks with 200 μg of MOG35–55 emulsion to induce EAE by active immunization. The emulsion was prepared under sterile conditions using MOG_35–55_ (85% purity; Espikem) in 300 μl of complete Freund’s adjuvant (CFA; Difco) containing Mycobacterium tuberculosis (8 mg/ml, strain H37Ra; Difco) and emulsified with phosphate buffer solution (PBS). All animals were injected with 500 ng of pertussis toxin (Sigma) intravenously on the day of immunization and 2 days later. Control animals received the same treatment as EAE mice without the immunogen MOG peptide, including complete CFA and pertussis toxin (referred to hereafter as “CFA”). Animals were daily scored for clinical symptoms of EAE according to the following scale: 0 = no clinical signs, 1 = flaccid tail, 2 = hindlimb weakness, 3 = hindlimb paresis, 4 = complete bilateral hindlimb paralysis, and 5 = death due to EAE; intermediate clinical signs were scored by adding 0.5. For each animal, the onset day was recorded as the day post-immunization (dpi) when it showed the first clinical manifestations.

### Laquinimod formulation for minipump and surgery (in vivo experiments)

Laquinimod (Teva Pharmaceutical Industries, Netanya, Israel) was dissolved in 0.9% NaCl. One week before immunization, mice were implanted with subcutaneous osmotic minipumps allowing continuous intracerebroventricular (icv) infusion of either vehicle (vhl) or laquinimod (1.25 mg/kg/day) for 4 weeks [25, 27].

### Electrophysiology

Mice were killed by cervical dislocation, and cerebellar parasagittal slices (210 μm) were prepared from fresh tissue blocks of the brain using a vibratome. After 1 h of recovery time in a chamber containing oxygenated artificial cerebrospinal fluid (ACSF), single slices were transferred to a recording chamber and submerged in a continuously flowing ACSF at 2–3 ml/min gassed with 95% O_2_–5% CO_2_. The composition of the ACSF was (in mM): 126 NaCl, 2.5 KCl, 1.2 MgCl_2_, 1.2 NaH_2_PO_4_, 2.4 CaCl_2_, 11 glucose, and 25 NaHCO_3_. Purkinjie cells (PCs) could be easily identified using an Olympus BX50WI upright microscope with a ×40 water-immersion objective combined with an infrared filter. Whole-cell patch-clamp recordings were made with borosilicate glass pipettes (1.8 mm outer diameter; 2–5.5 MΩ) in voltage-clamp mode at the holding potential of − 70 mV. To detect spontaneous excitatory postsynaptic currents (sEPSCs), the recording pipettes were filled with internal solution containing the following (in mM): 125 K+-gluconate, 10 NaCl, 1.0 CaCl2, 2.0 MgCl2, 0.5 BAPTA, 10 HEPES, 0.3 GTP, 3.0 Mg-ATP, adjusted to pH 7.3 with KOH. Bicuculline (10 μM) was added to the external solution to block GABAA-mediated transmission. Laquinimod was added in the bath solution at final concentration of 30 μM for 2 h before recordings. Some experiments were performed in the presence of the GLT-1 inhibitor DHK (200 μM).

Spontaneous synaptic events were stored using P-CLAMP 10 (Molecular Devices) and analyzed offline on a personal computer with Mini Analysis Version 6.0.7 software (Synaptosoft). The detection threshold of spontaneous and miniature excitatory events was set at twice the baseline noise. Positive events were confirmed by visual inspection for each experiment. Analysis was performed on spontaneous synaptic events recorded during a fixed time epoch (1–2 min) sampled every 2 or 3 min. Only cells that exhibited stable frequencies and amplitudes were taken into account. For sEPSC kinetic analysis, events with peak amplitude between 5 and 40 pA were grouped, aligned by half-rise time, and normalized by peak amplitude. In each cell, all events between 5 and 40 pA were averaged to obtain rise times, decay times, and half widths.

### Ex vivo experiments

Cerebellar slices from 21 dpi EAE mice were incubated in oxygenated ACSF in the presence of vehicle (vhl) or laquinimod (30 μM) for 2 h. For each cerebellum, both experimental group (control and laquinimod) were included. Each slice was quickly dried and then snap frozen in dry ice. Four animals were used for protein extraction and western blot, and the same number of animals were employed for mRNA extraction and quantitative real-time PCR (qPCR).

### Western blot (WB)

Twenty-two dpi cerebella from EAE-vhl and EAE-laquinimod mice were isolated and snap frozen after sacrifice of the animals by cervical dislocation. Slices from ex vivo experiments and whole cerebella were next lysed in RIPA buffer supplemented with protease inhibitors. Protein quantification and western blot condition as in [25].

The following primary antibodies were used: mouse anti-β-actin (1:20,000; Sigma) for 1 h at RT; rabbit anti-GLAST/EAAT1 (1:5000; Abcam) 30 min at RT; mouse anti-glial fibrillary acidic protein (GFAP) (1:4000; Immunological Science) overnight at + 4 °C; guinea pig anti-GLT-1 (1:10,000; Millipore) 1 h at RT. Membranes were incubated with secondary HRP-conjugated IgG anti-rabbit (1:5000 for 30 min at RT), anti-mouse (1:4000 and 1:10,000 1 h at RT for GFAP and β-actin, respectively), and anti-guinea pig (1:10,000 1 h at RT) (all from Amersham GE Healthcare, formerly Amersham Biosciences). Immunodetection was performed by ECL reagent (Amersham GE Healthcare, formerly Amersham Biosciences), and membrane was exposed to film (Amersham GE Healthcare, formerly Amersham Biosciences). Densitometric analysis of protein levels was performed with ImageJ software (https://imagej.nih.gov/ij/). WB results were presented as data normalized to control CFA values.

### RNA extraction and qPCR

Twenty-two dpi cerebella from EAE-vhl and EAE-laquinimod mice were dissected in RNAse-free conditions. Total RNA was extracted according to the standard miRNeasy Micro kit protocol (Qiagen) from both cerebella (in vivo experiments) and cerebellar slices (ex vivo experiments). Next, 700–1650 ng of total RNA were reverse-transcribed using High-Capacity cDNA Reverse Transcription Kit (Applied Biosystems), and 10–50 ng of complementary DNA (cDNA) were amplified in triplicate using the Applied Biosystem 7900HT Fast Real-Time PCR system. SensiMix SYBR Hi-Rox Kit (Bioline) was utilized for the quantification of messenger RNA (mRNAs) coding for ionized binding protein type-1 (IBA-1), glial fibrillary acidic protein (GFAP), GLAST, GLT-1, and BDNF by using the following primers:

- Aif1 mRNA coding for IBA-1 (NM_019467): forward GACAGACTGCCAGCCTAAGACAA; reverse CATTCGCTTCAAGGACATAATATCG
- Gfap (NM_010277.3): forward ACATCGAGATCGCCACCTACAG; reverse CCTCACATCACCACGTCCTTG
- Slc1a3 mRNA coding for GLAST protein (NM_148938.3): forward GCAGTGGACTGGTTTCTGGACC; reverse ACGGGTTTCTCCGGTTCATT
- Slc1a2 mRNA coding for GLT-1 protein (NM_001077515.2): forward CGGGATGAACGTCTTAGGTCTG; reverse ATGATGAGGCCCACGATCAC
- Bdnf (NM_007540): forward ACCATAAGGACGCGGACTTGT; reverse AAGAGTAGAGGAGGCTCCAAAGG
- Actb (NM_007393.3): forward CCTAGCACCATGAAGATCAAGATCA; reverse AAGCCATGCCAATGTTGTCTCT

For the mRNA quantification of Cd3, Il1b, and M1 (Cd86, MHC-II, CD16, and iNOS) and M2 (Arg1, Ym1, FIZZ1, and Tgfb1) markers, SensiMix II Probe Hi-Rox Kit (Bioline; Meridian Life Science) and the following TaqMan gene expression assays were used:

- Cd3e ID: Mm00599684_g1;
- Il1b ID: Mm00434228_m1;
- Cd86 ID: Mm00444543_m1;
- H2Ab1 (coding for a component of MHC-II) ID: Mm00439216_m1;
- Fcgr3 (coding for CD16) ID: Mm00438882_m1;
- Nos2 (coding for iNOS) ID: Mm00440502_m1;
- Arg1 ID: Mm00475988_m1;
- Chil3 (coding for Ym1) ID: Mm00657889_mH;
- Retnla (coding for FIZZ1) ID: Mm00445109_m1;
- Tgfb1 ID: Mm01178820_m1;
- Actb ID: Mm00607939_s1.

For both SYBR and TaqMan qPCR experiments, mRNA relative quantification was performed using the comparative cycle threshold (2^−ΔΔCt^) method. β-actin was used as endogenous control. All data are expressed relative to EAE-vhl.

miR-142-3p expression was evaluated using miR-142-3p TaqMan miRNA assay (catalog ID 000464) and TaqMan miRNA Reverse Transcription Kit according to the manufacturer’s instructions (Applied Biosystems). Each reaction of amplification was performed in triplicates with SensiMix SYBR II Probe Hi-Rox Kit (Bioline, Meridian Life Science); data, normalized to U6B snRNA (catalog ID 001973) and control samples (EAE-vhl), are represented as 2^−ΔΔCt^.

### Immunofluorescence (IF) and confocal microscopy

IF experiments were performed on slices taken from mice sacrificed at the peak of the EAE from at least two different immunization experiments, similarly to [25]. Animals were deeply anesthetized and perfused intracardially with ice-cold 4% paraformaldehyde (PFA).

For ex vivo experiments, after incubation with laquinimod or vhl, cerebellar slices (200 μm) were fixed in 4% PFA and equilibrated with 30% sucrose before cutting 30 μm slices. The following primary antibodies were used overnight at 4 °C in Triton X-100 0.25%: rabbit anti-GFAP (1:500; Dako) and guinea-pig anti-GLT-1 (1:5000; Millipore). AlexaFluor-488-conjugated donkey anti-rabbit (1:200; Invitrogen) and Cy3-conjugated donkey anti-guinea pig (1:200; Jackson ImmunoResearch Laboratories) were used as secondary antibodies. Nuclei were stained with DAPI. All images were acquired using an LSM7 Zeiss confocal laser-scanner microscope (Zeiss) with a ×20 (zoom ×1 or ×2) objective. All images had a pixel resolution of 1024 × 1024. The confocal pinhole was kept at 1.0, the gain and the offset were lowered to prevent saturation in the brightest signals, and sequential scanning for each channel was performed. Images were exported in Tiff format and adjusted for brightness and contrast as needed using ImageJ software.

### Statistical analysis

For each type of experiment, at least three mice per group were employed. Throughout the text, “*n*” refers to the number of animals, except for electrophysiology, where it means the number of cells, and ex vivo experiments of WB and qPCR. Data were presented as the mean ± SEM. The significance level was established at *p* < 0.05. Statistical analysis was performed using unpaired Student’s *T* test for comparisons between two groups and non-parametric Mann–Whitney test, where needed. Multiple comparisons were analyzed by one-way ANOVA for independent measures followed by Tukey’s HSD.

## Results

### icv administration of laquinimod ameliorates motor disability of EAE mice and upregulates Slc1a3 mRNA coding for GLAST

**Fig. 1:**
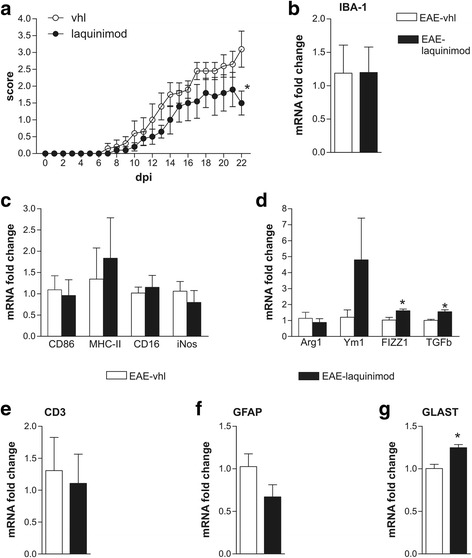
Central delivery of laquinimod attenuates motor disability in the acute phase of the disease without affecting cerebellar inflammation. **a** Clinical score of EAE mice treated by icv injection of laquinimod (1.25 mg/kg/day for 4 weeks) was attenuated at the peak of the acute phase in comparison with EAE-vhl (Mann–Whitney test *p < 0.05). **b**–**f** Quantification of several inflammatory markers performed by qPCR in the EAE cerebellum. Bar graphs show that mRNA coding for IBA-1 (**b**) and M1-like markers (**c**) were not modified by laquinimod treatment, while there was an increase of M2-like markers Retnla (Ym1) and Tgfb (**d**). CD3 (**e**) and GFAP (**f**) mRNA were unchanged between EAE and EAE-laquinimod mice while Slc1a3 mRNA coding for GLAST (**g**) was increased in EAE-laquinimod mice (unpaired *T* test *p < 0.05). All data are represented as mean ± SEM. qPCR results are expressed as fold change of EAE-vhl samples

To address a direct neuroprotective effect of laquinimod in EAE mice, we delivered the drug icv for 4 weeks by means of osmotic minipumps, starting 1 week before EAE induction. This preventive treatment did not elicit significant changes in daily clinical score of the treated animals compared with the vhl group in the first, inflammatory phases of the disease (Fig. 1a, *n* = 5 EAE-vhl, *n* = 5 EAE-laquinimod 1.25 mg/kg/day; Mann–Whitney non-parametric test *p* > 0.05). Also, the day onset was unchanged between the groups (EAE-vhl 10.6 ± 1.29 days vs EAE-laquinimod 1.25 mg/kg/day 11.4 ± 1.21 day, unpaired *T* test *p* > 0.05). However, laquinimod resulted in significant amelioration of motor disability in the late stage of EAE, when neurodegenerative damage was more pronounced (Mann–Whitney non-parametric test *p* < 0.05 at 22 dpi).

Next, we used qPCR to assess the effect of laquinimod on the inflammatory reaction of the cerebellum in treated animals taken at 22 dpi, when neuroinflammation and glutamatergic alterations are exacerbated pathological processes in EAE. To this end, we analyzed several markers of inflammation, microgliosis, T cell infiltration, and astrogliosis [25, 28]. As shown in Fig. 1b, the Aif1 mRNA coding for IBA-1, marker of macrophage/microglia, was not different between EAE–vhl and EAE–laquinimod groups (fold change: EAE–vhl 1.18 ± 0.42 *n* = 3; EAE–laquinimod 1.19 ± 0.38 *n* = 4; unpaired *T* test *p* > 0.05). To better investigate the effect of laquinimod on microglia cells, we also analyzed the expression of M1- and M2-like microglia-specific markers by means of qPCR [29]. The following M1-like markers were investigated: CD86 mRNA coding for a costimulatory protein for antigen presentation functions of microglia; H2ab1 mRNA coding for MHC-II component; Fcgr3 mRNA coding for CD16 protein, required for antibody recognition; and Nos-2 mRNA coding for iNos, a protein involved in immune response. None of these markers were significantly affected by laquinimod treatment (Fig. 1c; CD86 fold change: EAE–vhl 1.093 ± 0.33, EAE–laquinimod 0.96 ± 0.37; H2ab fold change: EAE–vhl 1.346 ± 0.73, EAE–laquinimod 1.835 ± 0.95; Fcgr3 fold change: EAE–vhl 1.018 ± 0.14, EAE–laquinimod 1.151 ± 0.28; Nos-2 fold change: EAE–vhl 1.06 ± 0.23, EAE–laquinimod 0.797 ± 0.28; unpaired *T* test *p* > 0.05 for each marker, EAE–vhl *n* = 3, EAE–laquinimod *n* = 4). Furthermore, we checked the expression of four M2-like markers: Arg1 mRNA coding for a protein involved in matrix deposition; Chil3 mRNA coding for Ym1 protein, a macrophage enzyme proposed to prevent extracellular matrix degradation; Retnla mRNA coding for FIZZ1 protein, which promotes deposition of extracellular matrix; and Tgfb with a number of functions in immune system regulation. Interestingly, Retnla and Tgfb were significantly upregulated in EAE–laquinimod cerebellum, suggesting that the upregulation of M2-like-specific markers might contribute to promote central beneficial effects of the drug (Fig. 1d; Arg1 fold change: EAE–vhl 1.132 ± 0.38; EAE–laquinimod 0.875 ± 0.24, unpaired *T* test *p* > 0.05; Chil3 fold change: EAE-vhl 1.2 ± 0.46; EAE-laquinimod 1.151 ± 0.28, unpaired *T* test *p* > 0.05; Retnla fold change: EAE-vhl 1.026 ± 0.17; EAE-laquinimod 1.608 ± 0.11, unpaired *T* test *p* < 0.05; Tgfb fold change: EAE-vhl 1.006 ± 0.007; EAE-laquinimod 1.547 ± 0.12, unpaired *T* test p < 0.05; EAE-vhl *n* = 3, EAE-laquinimod *n* = 4).

Regarding the expression of the T cell marker CD3, we did not observe any difference between the two experimental groups, indicating that the treatment did not affect T cell infiltration in the cerebellum (Fig. 1e; fold change: EAE-vhl 1.30 ± 0.52 *n* = 3, EAE-laquinimod 1.10 ± 0.46 *n* = 4; unpaired *T* test *p* > 0.05). Moreover, the mRNA of the glial marker, GFAP, was not modulated by laquinimod (Fig. 1f; fold change: EAE-vhl 1.02 ± 0.15 *n* = 3, EAE-laquinimod 0.67 ± 0.14 *n* = 4; unpaired *T* test *p* > 0.05). Taken together, these data indicate that the preventive and central laquinimod treatment did not induce a prominent effect on inflammation in the cerebellum of EAE mice.

Lastly, we investigated the expression of the Slc1a3 mRNA coding for the glutamate transporter GLAST. Laquinimod upregulated Slc1a3 mRNA (Fig. 1g; fold change: EAE-vhl 1.00 ± 0.05 *n* = 3, EAE-laquinimod 1.24 ± 0.04 *n* = 4; unpaired *T* test *p* < 0.05), suggesting a possible effect of laquinimod on glutamatergic transmission in EAE cerebellum.

### Acute incubation of laquinimod on EAE cerebellar slices recovers glutamatergic synaptic alterations

**Fig. 2:**
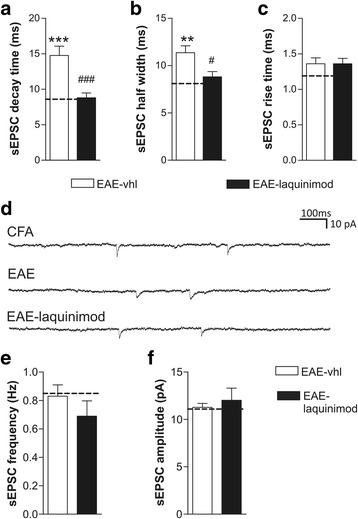
Acute incubation of laquinimod protects against EAE-induced excitotoxicity. **a**–**c** Whole-cell patch-clamp recordings from PCs show that bath incubation of cerebellar slices with laquinimod (30 μM, 2 h) promoted a beneficial effect on EAE-induced alteration in terms of half width (**a**) and decay time (**b**). Other parameters of the sEPSCs, like rise time (**c**), frequency (**e**), and amplitude (**f**), were unchanged among groups. **d** The electrophysiological traces are examples of sEPSCs recorded from control (CFA), EAE-vhl and EAE-laquinimod slices. Dashed lines in the graphs represent control CFA values. Data are expressed as mean ± SEM. One-way ANOVA post hoc comparisons: EAE-vhl vs CFA control **p < 0.01, ***p < 0.001; EAE-laquinimod vs EAE-vhl #*p* < 0.05, ###*p* < 0.001

Laquinimod is a small molecule able to passively cross the blood–brain barrier (BBB) that has been shown to rapidly reach the brain in both physiological and pathological conditions such as EAE [19]. To better investigate the direct effect of laquinimod on glutamatergic excitotoxicity, we adopted an ex vivo model of incubation of laquinimod on cerebellar slices taken from EAE mice (21–25 dpi). After 2 h of laquinimod bath application (30 μM), we recorded spontaneous glutamatergic currents (EPSCs) from Purkinje cells (PCs). As previously reported [25], under the EAE conditions, the duration of EPSCs—decay time and half width—were significantly increased compared to control slices (Fig. 2a–d; EAE *n* = 9, decay time 14.76 ± 1.29 ms, half width 11.36 ± 0.72 ms; CFA *n* = 7, decay time 8.6 ± 0.71 ms, half width 8.10 ± 0.58 ms; one-way ANOVA Tukey post hoc analysis: EAE vs CFA decay time *p* < 0.001, half width *p* < 0.01). Interestingly, laquinimod was able to significantly recover the kinetic alterations of glutamatergic transmission in EAE cerebellum (EAE-laquinimod *n* = 14, decay time 8.805 ± 0.67 ms, half width 8.81 ± 0.56 ms; one-way ANOVA Tukey post hoc analysis: decay time EAE vs EAE-laquinimod *p* < 0.001, half width *p* < 0.05) (Fig. 2a–d). Rise time was unchanged among groups (Fig. 2c, d; EAE 1.36 ± 0.08 ms, CFA 1.09 ± 0.08 ms, EAE-laquinimod 1.36 ± 0.08 ms; one-way ANOVA *p* > 0.05). Furthermore, laquinimod incubation on EAE slices did not affect the frequency and the amplitude of sEPSC (frequency: CFA 0.85 ± 0.12 Hz, EAE 0.83 ± 0.08 Hz, EAE-laquinimod 0.69 ± 0.10 Hz; amplitude: CFA 11.10 ± 0.57 pA, EAE 11.27 ± 0.42 pA, EAE-laquinimod 12.02 ± 1.29 pA; one-way ANOVA *p* > 0.05; Fig. 2e, f). Altogether, these results suggest that laquinimod exerts a beneficial effect against EAE-induced postsynaptic alteration.

### The beneficial effect of laquinimod on glutamatergic transmission is independent of IL-1β-miR-142-3p-GLAST axis

**Fig. 3:**
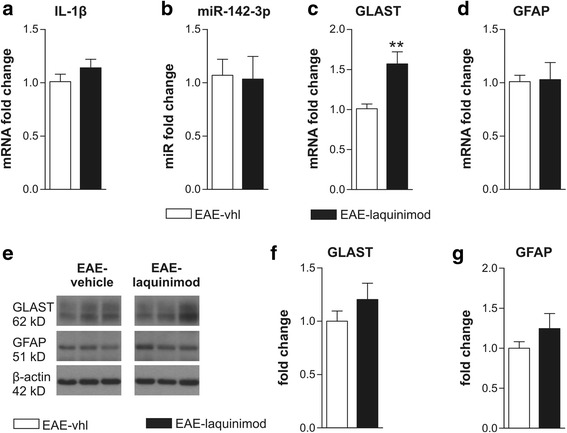
The anti-excitotoxic effect of laquinimod does not involve the IL-1β-miR-142-3p-GLAST regulatory axis. **a**–**d** qPCR experiments performed on EAE cerebellar slices incubated with or without laquinimod (30 μM, 2 h) show that laquinimod did not modulate IL-1β mRNA (**a**), miR-142-3p (**b**), and GFAP mRNA (d) while significantly upregulated Slc1a3 mRNA coding for GLAST (**c**). **e**–**g** WB analysis for GLAST (**e**, **f**) and GFAP (**e**, **g**) protein levels indicate that both proteins were unchanged after laquinimod treatment on EAE cerebellar slices. All data are expressed as mean ± SEM and as fold change of EAE-vhl samples. Unpaired *T* test: **p < 0.01

We speculated whether laquinimod beneficial effect on EAE synaptic alterations could be due to the interference with the excitotoxic mechanism involving the IL-1β-miR-142-3p-GLAST regulatory axis, recently described in [26]. Indeed, GLAST dysfunction in EAE cerebellum depends on a downregulation of the protein which is caused by an IL-1β-dependent upregulation of miR-142-3p, which targets the Slc1a3 mRNA coding for GLAST [25, 26]. Therefore, we first used qPCR to quantify the expression level of IL-1β mRNA, miR-142-3p, and Slc1a3 mRNA in EAE cerebellar slices incubated with 30 μM laquinimod compared with EAE-vhl slices. As shown in Fig. 3a, b, neither IL-1β transcript nor miR142–3p was corrected by laquinimod acute treatment (IL-1β fold change: EAE 1.07 ± 0.15, EAE-laquinimod 1.03 ± 0.21; miR-142-3p fold change: EAE 1.07 ± 0.15, EAE-laquinimod 1.03 ± 0.21; *n* = 7 per group; unpaired *T* test *p* > 0.05 for both comparisons), while we found a significant increase of Slc1a3 mRNA compared to vehicle slices (Fig. 3c; GLAST fold change: EAE-vhl 1.01 ± 0.063, EAE-laquinimod 1.57 ± 0.15; *n* = 7 per group; unpaired *T* test *p* < 0.01). As observed in the icv laquinimod experiments, Slc1a3 mRNA GLAST upregulation was not accompanied by significant changes in GFAP expression (Fig. 3d; GFAP fold change: EAE-vhl 1.01 ± 0.06, EAE-laquinimod 1.03 ± 0.16; *n* = 7 per group; unpaired *T* test *p* > 0.05).

To better characterize the involvement of GLAST in the laquinimod anti-excitotoxic effect, we used WB analysis to quantify GLAST and GFAP protein levels. Consistent with the qPCR quantification, GFAP protein content was unchanged between the two groups (Fig. 3e–g; fold change to EAE-vhl: EAE-vhl 1 ± 0.08 *n* = 13, EAE-laquinimod 1.24 ± 0.18, *n* = 14; unpaired *T* test, *p* > 0.05), suggesting that laquinimod did not affect astrogliosis in EAE cerebellar slices. Conversely, we could not detect significant differences in GLAST content between the two experimental groups (Fig. 3e, f; fold change to EAE-vhl: EAE-vhl 1 ± 0.09 *n* = 13, EAE-laquinimod 1.204 ± 0.152, *n* = 14; unpaired *T* test, *p* > 0.05). These data suggest that, although laquinimod induced an upregulation of the Slc1a3 mRNA, GLAST protein synthesis was impaired likely due the presence of high levels of miR-142-3p in EAE cerebellum.

Altogether, these results indicate that laquinimod recovers glutamatergic transmission through a mechanism that is not dependent on GLAST upregulation.

### Laquinimod induces GLT-1 upregulation in EAE cerebellar slices, likely leading to a recovery of glutamate alterations

The above results prompted us to investigate another possible mechanism of laquinimod-mediated reduced glutamate excitotoxicity. Although glutamate removal at tripartite synapse in the molecular layer of cerebellum is mediated mainly by GLAST, Bergmann glial (BG) cells express another glutamate transporter GLT-1, which is functionally less relevant and less expressed than GLAST in BG in healthy conditions [30]. We hypothesized that, under laquinimod administration, GLT-1 could compensate for GLAST dysfunction, thereby contributing to maintenance of proper glutamate homeostasis. To address this hypothesis, we first investigated GLT-1 expression in EAE condition and then the possible involvement of GLT-1 in laquinimod-mediated synaptic recovery.

**Fig. 4:**
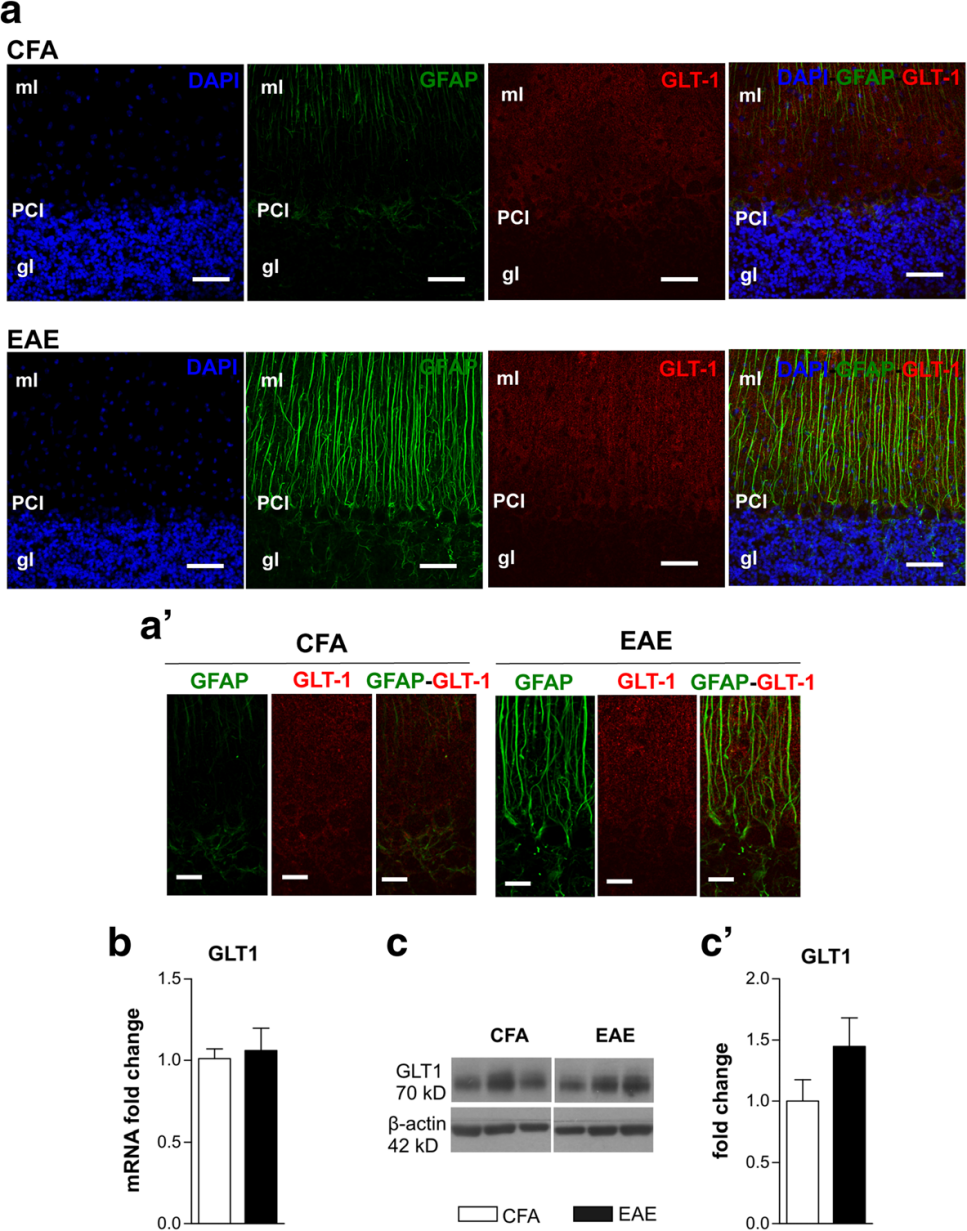
Cerebellar GLT-1 levels are unchanged during EAE. **a**, **a’** Confocal images of cerebellar parasagittal slices stained for GLT-1 (red), GFAP (green), and nuclei (blue) show that the intensity of GLT-1 was not different between CFA and EAE slices; a diffuse labelling is visible in the molecular layer (ml), the Purkinjie cell layer (PCl) and, although less intensely, the granular layer (gl). Scale bar 50 μm (**a**) and 20 μm (**a’**). Nuclei were stained with DAPI in blue. **b** The graph shows Slc1a2 mRNA coding for GLT-1 analyzed by qPCR performed in EAE and CFA cerebellar slices. **c**, **c’** Images of WB probed for GLT-1 and β-actin as internal control (**c**) and densitometric analysis of GLT-1 levels (**c’**) in EAE and CFA cerebellar slices. All data are expressed as mean ± SEM and as fold change of CFA samples

We assessed GLT-1 expression by performing both immunofluorescence experiments and western blot analysis in EAE cerebella taken at 22 dpi, in comparison to control CFA. As shown in Fig. 4a, cerebellar sagittal sections were immunostained for GFAP (green) and GLT-1 (red) to perform confocal imaging. As previously observed, GFAP staining was strikingly increased in EAE slices. Regarding GLT-1 protein, it localized in BG cells and astrocytes in the molecular layer (ml) and granular layer (gl) of the cerebellar cortex as reported by others [31]. In EAE cerebellar slices, GLT-1 staining seemed a bit more intense than CFA (Fig. 4a, a’). To better characterize GLT-1 expression, we assessed Slc1a2 mRNA coding for GLT-1 and protein content in 22 dpi EAE cerebellar lysates compared with experimental control CFA. We did not detect significant differences between the two groups in either Slc1a2 mRNA (Fig. 4a, b; fold change: CFA 1.01 ± 0.06, *n* = 7, EAE 1.06 ± 0.14, *n* = 8; unpaired *T* test *p* > 0.05) or GLT-1 protein (Fig. 4c, c’; fold change to CFA values: CFA 1 ± 0.17, *n* = 10, EAE 1.44 ± 0.23, *n* = 9; unpaired *T* test *p* > 0.05), despite a small but non-significant increase of the protein observed in EAE conditions.

**Fig. 5:**
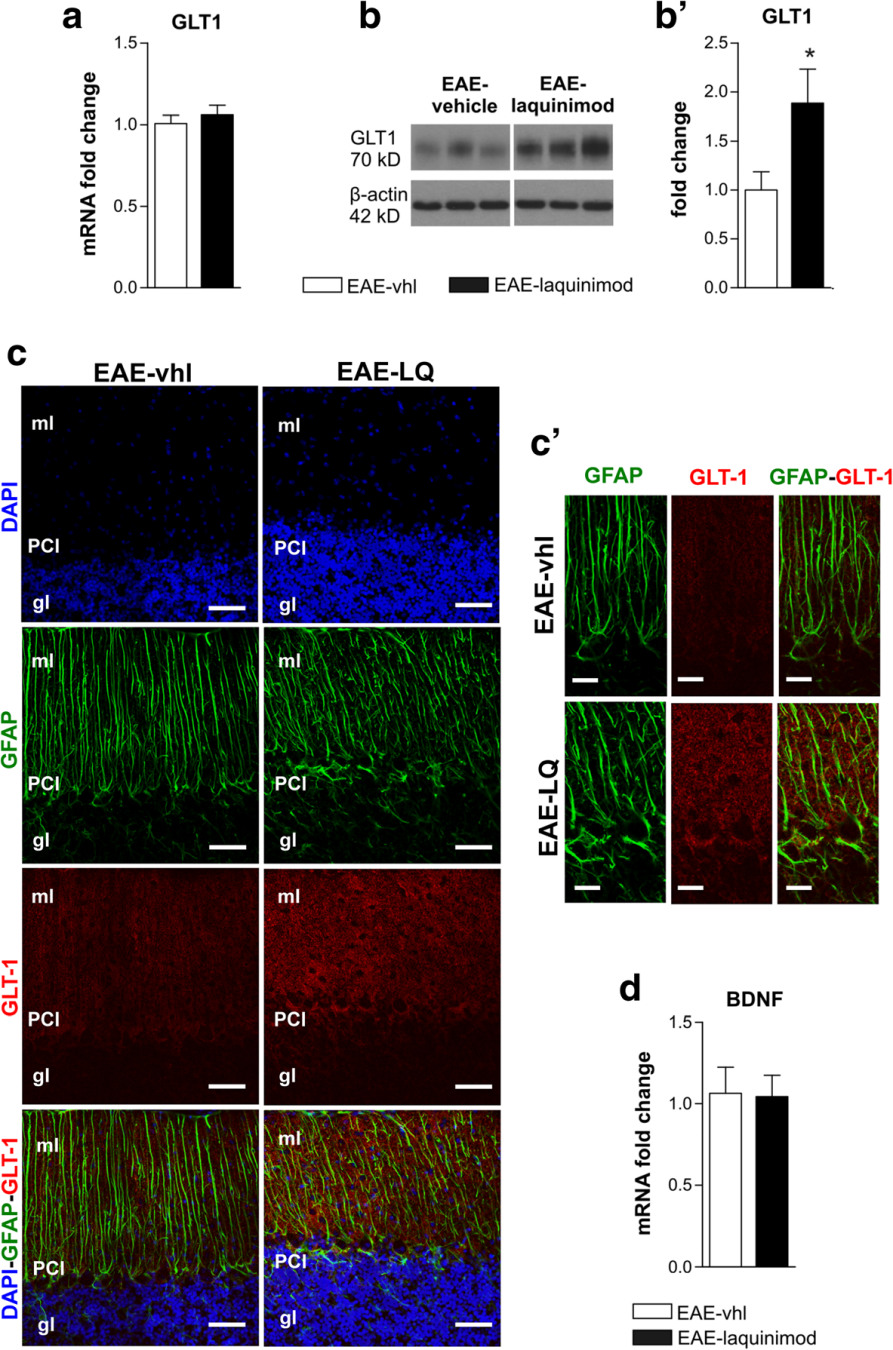
Laquinimod acute incubation on cerebellar slices significantly increases GLT-1. **a**–**b’** mRNA and protein analyses performed in EAE cerebellar slices incubated with or without laquinimod (30 μM, 2 h) indicate that laquinimod incubation did not change Slc1a2 mRNA (**a**) and significantly increased protein levels of GLT-1 (**b**, **b’**). **c** Immunostaining of cerebellar slices incubated with laquinimod or vhl shows that GLT-1 labelling (red) was increased in the molecular layer (ml) of EAE cerebellum in the presence of laquinimod compared to control slices (EAE-vhl), although the extent of astrogliosis was similar (green staining for GFAP). Scale bar 50 μm. High-magnification images in **c’** show GLT-1 staining pattern in EAE-vhl and EAE-laquinimod with a global increase of GLT-1 labelling around PC soma and in the ml. Scale bar 20 μm. Nuclei were stained with DAPI in blue. **d** The graph shows mRNA expression of the neurotrophin BDNF performed by qPCR in both experimental groups. All data are expressed as mean ± SEM and as fold change of CFA samples. Unpaired *T* test, *p < 0.05

We then analyzed the acute effect of laquinimod on GLT-1 expression and protein level in EAE cerebellar slices. Although Slc1a2 mRNA was unchanged between EAE-vhl and EAE-laquinimod slices (Fig. 5a; fold change: EAE-vhl 1.01 ± 0.05 *n* = 7, EAE-laquinimod 1.06 ± 0.06 *n* = 7, unpaired *T* test *p* > 0.05), a significant increase of the protein in the presence of laquinimod was observed (Fig. 5b, b’; fold change to EAE-vehicle: EAE-vhl 1 ± 0.18 *n* = 13, EAE-laquinimod 1.88 ± 0.34 *n* = 14; unpaired *T* test, *p* < 0.05). Based on these results, we used IF to investigate the effect of laquinimod on GLT-1 distribution in the cerebellar cortex (Fig. 5c). Astroglia activation was unaffected by laquinimod treatment, as shown by the comparable GFAP (green) staining in EAE and EAE-laquinimod sections. On the contrary, the overall intensity staining for GLT-1 was slightly increased in EAE slices treated with laquinimod (Fig. 5c, c’), corroborating WB findings.

These results point to GLT-1 as a putative target of laquinimod anti-excitotoxic action in the EAE cerebellum. Since laquinimod is reported to induce the expression of the neurotrophin, brain-derived neurotrophic factor (BDNF) in the brain of EAE mice [8] and BDNF positively regulates GluT expression [32]; we wondered whether BDNF could be involved in laquinimod-induced GLT-1 induction. We used qPCR to check the expression of BDNF in cerebellar slices exposed to laquinimod relative to vehicle slices, finding no differences between the two groups (Fig. 5d; fold change: EAE-vhl 1.06 ± 0.15 *n* = 7; EAE-laquinimod 1.04 ± 0.13 *n* = 7; unpaired *T* test *p* > 0.05) and suggesting that BDNF does not mediate the effect of laquinimod on GLT-1.

**Fig. 6:**
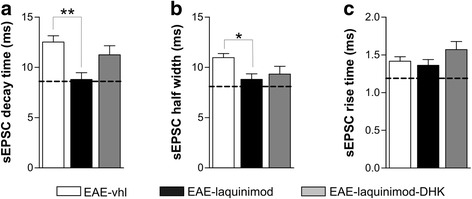
Laquinimod beneficial effect on glutamatergic transmission is mediated by GLT-1 function. **a**–**d** Bath application of the GLT-1 inhibitor (DHK) in EAE-cerebellar slices incubated with laquinimod blocked the anti-excitotoxic effect of laquinimod on decay time (**a**) and half width (**b**) on glutamatergic transmission (for comparison, the dashed line in the graphs indicates control values). **c** Rise time values were not significantly changed by any of the in vitro treatment. Electrophysiological events on the right are examples of sEPSCs recorded in each EAE condition (vhl, laquinimod and laquinimod plus DHK). Data are expressed as mean ± SEM. One-way ANOVA post hoc comparisons, *p < 0.05, **p < 0.01

To assess the contribution of GLT-1 to the functional recovery of glutamatergic transmission observed in EAE slices in the presence of laquinimod, we recorded sEPSC from PCs of EAE slices co-incubated with both laquinimod and the GLT-1 antagonist DHK. Under this experimental condition, we observed that the beneficial effect of laquinimod on sEPSC kinetic properties was largely prevented by the concomitant incubation with the GLT-1 antagonist DHK. As shown in Fig. 6a, decay time of sEPSC of EAE slices incubated with laquinimod was significantly reduced compared to both EAE-vhl and EAE-laquinimod -DHK (EAE-vhl *n* = 11, EAE-laquinimod *n* = 14, EAE-laquinimod -DHK *n* = 14; decay time: EAE-vhl 12.52 ± 0.617 ms, EAE-laquinimod 8.796 ± 0.670 ms, EAE-laquinimod -DHK 11.25 ± 0.899 ms; one-way ANOVA, Tukey post hoc analysis: EAE-vhl vs EAE-laquinimod *p* < 0.01). The same effect was observed when analyzing the half-width parameter of sEPSCs (Fig. 6b; half width: EAE-vhl 10.97 ± 0.40 ms; EAE-laquinimod 8.809 ± 0.55 ms; EAE-laquinimod -DHK 9.33 ± 0.78 ms; one-way ANOVA Tukey post hoc analysis: EAE-vhl vs EAE-laquinimod half width *p* < 0.05). Again, rise time values were unchanged among groups (Fig. 6c; rise time: EAE-vhl 1.416 ± 0.062 ms; EAE-laquinimod 1.362 ± 0.077 ms; EAE-laquinimod-DHK 1.57 ± 0.107 ms).

Taken together, these results indicate that laquinimod acute treatment ameliorates glutamatergic transmission in EAE cerebellum by inducing GLT-1 expression and improving its function.

## Discussion

In the present study, we identified a novel pathway through which laquinimod can exert a direct neuroprotective role in the CNS of EAE mice and likely in MS. We showed that laquinimod is able to ameliorate cerebellar glutamatergic transmission when directly incubated on EAE cerebellar slices and it exerts beneficial effect on clinical measures when delivered directly into the brain. We propose that laquinimod, which is able to cross the BBB, increases the expression of the glial GluTs at the tripartite synapse when it enters the CNS, leading to a recovery of the synaptic alterations. Mechanistically, laquinimod induces an upregulation of the Slc1a3 mRNA coding for GLAST and an upregulation of the GLT-1 protein which in turn attenuates excitotoxicity. Astrogliosis and the regulatory axis IL-1β /miR-142-3p, which impairs GLAST protein synthesis, seem to be unaffected by the treatment. We suggest that the recovery of glutamatergic transmission in EAE cerebellum is mainly mediated by GLT-1 overexpression and function.

We previously demonstrated a neuroprotective effect of laquinimod following subcutaneous daily injection in EAE mice. In particular, we showed that both preventive and therapeutic treatment fully prevented the alterations of GABAergic synapses in EAE striatum, the first limiting also the glutamatergic synaptic alterations [14]. In the present study, we investigated its potential neuroprotective effect on the regulatory axis IL-1β-miR-142-3p-GLAST, responsible for the synaptopathy, which affects the EAE cerebellum and likely MS brain.

In the MS pathophysiology, elevated levels of glutamate in the CSF of MS patients have been associated with excitotoxic damage of both neurons and oligodendrocytes [26]. Accordingly, decreased levels of GluT proteins and/or mRNAs have been observed in several brain areas of both EAE [25, 33–36] and MS [21, 37, 38]. Of note, reduced expression and function of these transporters have been reported in other neurological disorders, including amyotrophic lateral sclerosis, Alzheimer’s disease and Parkinson’s disease [39–43], suggesting common mechanisms of dysfunction of these transporters leading to excitotoxic damage. For this reason, to date, an extensive effort has been made not only to clarify the mechanism of downregulation of GluTs in these diseases but also to identify molecular targets for enhancement of GLAST and GLT-1 expression as a potential therapeutic approach [44]. In the present study, we demonstrated for the first time that both GLT-1 and GLAST are molecular targets of laquinimod with protective effects in EAE mice and potentially in MS. In particular, we demonstrated that the anti-excitotoxic activity of laquinimod was likely mediated by GLT-1, since pharmacological inhibition of this GluT blocked laquinimod beneficial effects on glutamatergic currents in ex vivo experiments. Therefore, we suggest that laquinimod, by inducing GLT-1 protein expression, potentiates GLT-1 function that in normal condition is negligible [30]. Notably, previous studies based on immunogold electron microscopy have shown that in the cerebellum GLT-1, like GLAST, is expressed in BG [31], leading to the notion that they are both glial-specific GluTs. However, based on mounting evidence of GLT-1 neuronal expression in other brain areas, like the hippocampus, we cannot rule out the contribution of neuronal GLT-1 to glutamatergic transmission in EAE-cerebellum in the presence of laquinimod [45, 46].

Dysregulation of GLAST and GLT-1 expression and function can occur at multiple levels in several neurological diseases, from abnormal genetic coding to altered post-translational modifications. For example, genetic dysregulation of GLT-1, such as single nucleotide polymorphisms (SNPs) and aberrant mRNA splicing of GLT-1 are known to impair protein expression and function, and are linked to several neurological diseases [47, 48]. In MS patients, an A to C SNPs on −181 position of the GLT-1 promoter decreases GLT-1 expression and increases plasma glutamate levels during relapse [49]. We have recently demonstrated that GLAST is regulated at posttranscriptional level by miR-142-3p under inflammatory conditions in EAE brain and likely in MS. Furthermore, several pharmacological agents, such as ceftriaxone [50, 51] estrogen [52], tamoxifen [53] and riluzole [54] and neurotrophic factors (BDNS, PDGF, EGF, GDNF, etc.) increase GLT-1 and GLAST expression at the transcription level via activation of nuclear factor κB (NF-κB) [32, 50, 53]. On the other hand, negative regulatory mechanisms of these GluTs have been linked to the transcription factor yin yang 1 (YY1) [55] and the NF-κB signaling [56]. In this regard, it has been shown that TNF seems to switch signals that normally result in promoter activation to signals that suppress the GLT-1 promoter [32, 53, 57].

In the present study, we propose that laquinimod exerts a direct neuroprotective effect by altering the transcriptional and/or post-transcriptional regulation of GluTs. On the one hand, laquinimod induced a transcriptional upregulation of GLAST with no effect in terms of protein enhancement, likely due to the inhibitory action of miR-142-3p that is still high despite the presence of laquinimod. Since Slc1a3 mRNA coding for GLAST is downregulated in BG cells by overactivation of glutamate receptors such as AMPAR or mGluR, it might be suggested that laquinimod interferes with these pathways [58]. Conversely, we observed that laquinimod upregulates GLT-1 protein mainly through a posttranscriptional regulation. In this regard, it might be speculated an effect of laquinimod on GLT-1 regulation by RNA splicing [59]. The splicing variants of Slc1a2 mRNA containing a long 5′-UTR are indeed associated with increased GLT-1 protein expression in response to extracellular factors such as corticosterone and retinol [60]. We also hypothesized that regulation of BDNF levels might represent a point of convergence, considering that laquinimod treatment enhances BDNF expression and reduces CNS injury in EAE mice [8]. However, we could not observe an enhancement of BDNF mRNA under laquinimod treatment, implying the involvement of a different mechanism. It has been recently demonstrated that laquinimod activates AhR, which is necessary for its therapeutic efficacy in the MOG-induced EAE model of MS [9]. Interestingly, using bone marrow chimeras it was shown that loss of AhR in the immune system or its deletion within the CNS leads to the total or partial inhibition of the beneficial effect of laquinimod in EAE, respectively. This finding suggests that laquinimod pharmacological activity may be partially dependent on the expression of AhR within the CNS, considering that expression of AhR within astrocytes limits CNS inflammation [61].

Finally, both in vivo and ex vivo experiments indicate that laquinimod showed negligible or null effects on inflammatory reaction typical of EAE. Astrogliosis, microgliosis and CD3 infiltration in the EAE cerebellum were unaffected by icv laquinimod treatment. However, laquinimod increased the expression of the M2-like markers Retnla and Tgfb with no effect on the expression of typical M1-like molecules, suggesting a negligible switch from M1- to M2-like phenotype. On the other hand, considering that the distinction of M1- and M2-like phenotypes of microglia more likely describes a conventional simplification than the actual state of microglia [62], we suggest that the upregulation of M2-like markers in EAE microglia cells might contribute to promote central beneficial effects of the drug.

## Conclusions

In conclusion, the present work supports the notion of a direct neuroprotective effect of laquinimod in the CNS that is likely independent of its anti-inflammatory action, highlighting a beneficial effect on the glutamatergic excitotoxic damage typical of EAE and MS brain, mediated by GLT-1 in astroglial cells.

## Abbreviations

ACSF: Artificial cerebrospinal fluid
AhR: Aryl hydrocarbon receptor
BDNF: Brain-derived neurotrophic factor
BG: Bergmann glia
cDNA: Complementary DNA
CFA: Complete Freund’s adjuvant
CNS: Central nervous system
CSF: Cerebrospinal fluid
dpi: Day post-immunization
EAAT1: Excitatory amino acid transporter
EAE: Experimental autoimmune encephalomyelitis
gl: Granular layer
GLAST: Glutamate–aspartate transporter
GLT-1: Glutamate transporter 1
GluT: Glutamate transporter
icv: Intracerebroventricular
IF: Immunofluorescence
MOG: Myelin oligodendrocyte glycoprotein
MS: Multiple sclerosis
PBS: Phosphate buffer solution
PC: Purkinje cell
PFA: Paraformaldehyde
qPCR: Quantitative real-time PCR
sEPSC: Spontaneous excitatory postsynaptic current
SNP: Single nucleotide polymorphism
vhl: Vehicle
